# A case of *chlamydia psittaci* caused severe pneumonia and meningitis diagnosed by metagenome next-generation sequencing and clinical analysis: a case report and literature review

**DOI:** 10.1186/s12879-021-06205-5

**Published:** 2021-06-30

**Authors:** Yunfeng Shi, Junxian Chen, Xiaohan Shi, Jiajia Hu, Hongtao Li, Xiaojie Li, Yanhong Wang, Benquan Wu

**Affiliations:** 1 Medical Intensive Care Unit, Department of Respiratory and Critical Care Medicine, Third Affiliated Hospital of Sun Yat-Sen University, Guangzhou, 510630 China; 2 Institute of Respiratory Diseases, Sun Yat-Sen University, Guangzhou, China; 3 Department of Laboratory Medicine, Third Affiliated Hospital of Sun Yat-Sen University, Guangzhou, 510630 China

**Keywords:** Psittacosis, *Chlamydia psittaci*, Severe pneumonia, Meningitis, Metagenome next-generation sequencing, Case report

## Abstract

**Background:**

Psittacosis, which is also known as parrot fever, is *Chlamydia psittaci (C. psittaci)* caused infectious disease*.* The clinical manifestations vary from asymptomatic infection to severe atypical pneumonia or even fatal meningitis. Early recognition of psittacosis is difficult because of its nonspecific clinical manifestations. Culture and gene probe techniques for *C. psittaci* are not available for routine clinical use, which makes the diagnosis difficult too. Although psittacosis has increasingly been recognized and reported in recent years, cure of severe pneumonia complicated with meningitis, with etiologic diagnosis aided by the use of metagenomic next-generation sequencing (mNGS), is still uncommon. So, it is necessary to report and review such potentially fatal case.

**Case presentation:**

This report describes a 54-year-old woman with *C. psittaci* caused severe atypical pneumonia and meningitis. She presented with symptoms of fever, dry cough and dyspnea, accompanied by prominent headache. Her condition deteriorated rapidly to respiratory failure and lethargy under the treatment of empirical antibacterial agents, and was treated with invasive mechanical ventilation soon. She denied contact with birds, poultry or horses, but unbiased mNGS of both the bronchoalveolar lavage fluid (BALF) and the cerebrospinal fluid (CSF) identified sequence reads corresponding to *C. psittaci* infection, and there was no sequence read corresponding to other probable pathogens. Combined use of targeted antimicrobial agents of tetracyclines, macrolides and fluoroquinolones was carried out, and the patient’s condition improved and she was discharged home 28 days later. Her status returned close to premorbid condition on day 60 of follow-up.

**Conclusions:**

When clinicians come across a patient with atypical pneumonia accompanied by symptoms of meningitis, psittacosis should be taken into consideration. mNGS is a promising detection method in such condition and is recommended.

**Supplementary Information:**

The online version contains supplementary material available at 10.1186/s12879-021-06205-5.

## Background

Human psittacosis, also known as parrot fever or ornithosis, is a zoonotic infectious disease whose agent is the obligate intracellular bacterium *Chlamydia psittaci* (*C. psittaci*). Although psittacosis is not a common disease, it has been reported worldwide, including China, USA, Europe, and especially in Australia [1–4]. It accounts for 1–2% of cases of community-acquired pneumonia (CAP) annually [5]. However, the clinical manifestations vary from asymptomatic infection to fatal systemic illness [3]. There is no convenient and specific test for identification of *C. psittaci*. In recent years, with the improvement in detection methods and understanding of the disease, increased cases of psittacosis have been reported. Here, we present a successfully cured case of severe pneumonia and meningitis caused by *C. psittaci*, with etiologic diagnosis aided by the use of metagenomic next-generation sequencing (mNGS). To the best of our knowledge, this is the first case of such infection caused by *C. psittaci* and detected by mNGS in time with good outcome.

## Case presentation

The patient was admitted to our hospital because of recurrent headache and fever for 20 d and dyspnea for 3 d. She presented with headache 20 d before admission, which consisted of tolerable fluctuating pain in the left occipital region, which last 2–3 min every attack and resolved naturally. Low-grade fever (unmeasured temperature) was present from the next day, accompanied by sore throat and dry cough without sputum. There was no chills, muscle soreness or limb weakness. After oral drug treatment (unknown) prescribed by local hospital, sore throat and dry cough were relieved temporarily. Fever recurred at 15 d before admission with a temperature of 38 °C, accompanied by obvious fatigue, dry cough, nausea and non-projectile vomiting of gastric contents three or four times per day. About 1 w before admission, the patient suffered long-term memory loss, bilateral hearing loss and limb weakness. Urinary incontinence occurred on the next day, without syncope, loss of consciousness, and general tics. Three days before admission, the patient had dyspnea, and her previous symptoms became aggravated, so she was sent immediately to the emergency department of the Third Affiliated Hospital of Sun Yat-Sen University. During the progress of routine examination in the emergency department, her condition deteriorated rapidly to severe hypoxemia and lethargy. After tracheal intubation and ventilator assisted ventilation, she was transferred to the medical intensive care unit (MICU) for further treatment.

The patient suffered from hypothyroidism for 5 years and regularly took oral levothyroxine (Euthyrox 25 mg/d). She also had hepatitis B and was treated with entecavir 0.5 mg/d regularly. Her thyroid function and liver function were normal during regular out-patient visit. She had no diabetes mellitus, head trauma, or psychiatric or psychological disease. She had no tuberculosis or lung cancer. She is neither a smoker nor a drug addict. She resides in Guangdong province, southern China. She had no recent travel, tick bites, or acute upper respiratory infection. She had not been in contact with any birds, poultry or horses directly. She didn’t shop at live-bird markets, nor had friends or relatives with pet birds.

When the patient was admitted to MICU (day 1), her vital signs were as follows: body temperature 39.0 °C, pulse rate 128 beats/min, respiratory rate 20 breaths/min, blood pressure 105/78 mmHg with administration of dopamine 6 μg/(kg·min) via micro pump, and pulse oxygen saturation 96.4% with a fraction of inspired oxygen (FiO_2_) of 0.90. The patient presented with lethargy while under treatment with sedation. The rough breath sounds and wet rales on both lungs were heard on auscultation. There was no audible murmur on cardiac auscultation. Tenderness and hepatosplenomegaly were not detected. She had no neck stiffness. Kernig sign was suspicious positive. No rash was observed.

**Fig. 1:**
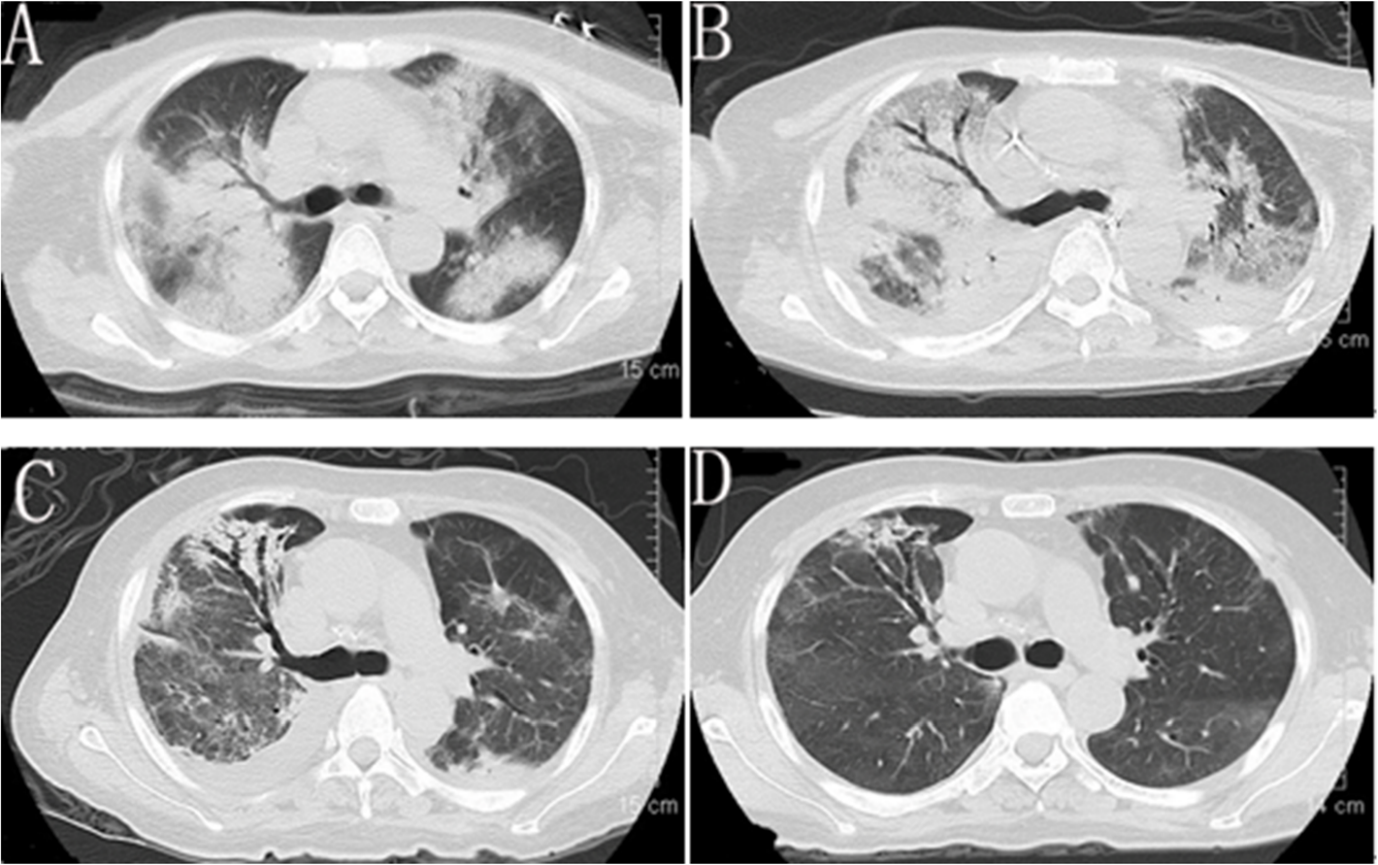
Chest computed tomography scan at admission (**A**), 14d later (**B**), 22d later (**C**) and 47d later (**D**)

**Fig. 2:**
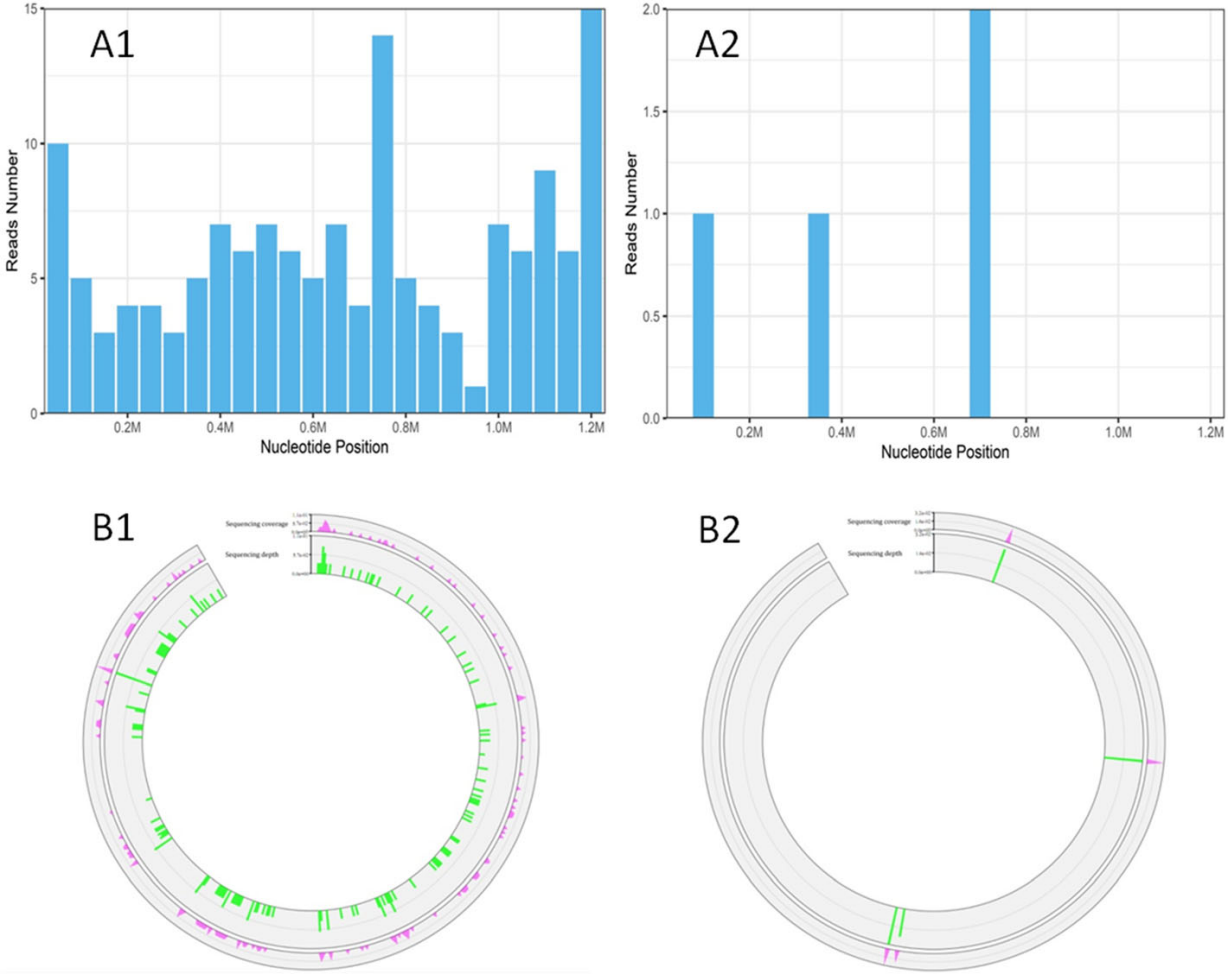
The cover charts (**A1, A2**) and Sequencing coverage and depth (**B1, B2**) of *Chlamydia psittaci* in bronchoalveolar lavage fluid (**A1, B1**) and cerebrospinal fluid (**A2, B2**)

Laboratory data upon admission to MICU revealed white blood cell (WBC) count 6.37 × 10^9^/L with an elevated neutrophil ratio of 94.7%. The concentration of C-reactive protein (CRP) and procalcitonin (PCT) were 175.0 mg/L and 6.87 ng/mL, respectively. Erythrocyte sedimentation rate (ESR) was 65 mm/h. Lactate was 1.3 mmol/L. Electrolytes and creatinine were within normal limits. Albumin level was 29.4 g/L. Aspartate aminotransferase (AST) was 218 U/L and alanine aminotransferase (ALT) was 97 U/L. Arterial blood gas analysis showed a pH of 7.378, PO_2_ of 79.4 mmHg, PCO_2_ of 32.8 mmHg, and oxygenation index of 88.2. The result of interferon-gamma release assay was negative. Hepatitis B virus (HBV) infection indicators: HBV surface antigen (+), HBV e antibody (+), HBV core antibody (+), HBV-DNA 610 IU/mL. Tests for influenza A virus, influenza B virus, cytomegalovirus, Epstein Barr virus, *Toxoplasma*, rubella virus, and herpes simplex virus were all negative. Cryptococcal antigen was negative. Galacto Mannan test and β-D-Glucan test were both negative. All the indicators were negative for routine laboratory screening (anti-nuclear antibody, anti-extractable nuclear antigen antibody, and anti-neutrophilic cytoplasmic antibody, et al) for autoimmune diseases. The number of CD3, CD4 and CD8 T lymphocytes in blood was 76/mm^3^, 44/mm^3^ and 28/mm^3^ respectively. Lumbar puncture showed intracranial pressure of 212 mm H_2_O and CSF was transparent and colorless. CSF cytology and biochemistry showed WBC count 6 × 10^6^/L, red blood cell count 124 × 10^6^/L, protein 0.267 g/L (normal range 0.15–0.4 g/L), glucose 5.83 mmol/L (normal range 2.5–3.9 mmol/L), and chloride ions 125.9 mmol/L (normal range 121.0–129.0 mmol/L), adenosine deaminase (ADA) 1 U/L (normal value: < 40 U/L). Abdominal ultrasound examination found no abnormality. Chest computed tomography (CT) revealed multiple patchy shadows on both lungs and bilateral pleural effusion (Fig. 1A). Head CT scan showed no findings of acute infection or cerebral hemorrhage. After admission, results of two sets of peripheral blood cultures during episodes of fever were negative. Unbiased mNGS of the BALF identified 161 of 79,722 sequence reads corresponding to *C. psittaci*, there was no sequence read corresponding to other pathogens. Unbiased mNGS of the CSF identified four of 93,308 sequence reads corresponding to *C. psittaci* (Fig. 2). One sequence read corresponding to *candida parapsilosis*(*C. parapsilosis*) in CSF was detected. Clinical laboratory tests and imaging were performed by the department of laboratory and image center of the Third Affiliated Hospital of Sun Yat-sen University, respectively. mNGS was performed and reported by Guangzhou Kingmed Medical Test Center Co.Ltd.. Methods and quality control of mNGS see in Additional file 1.

**Fig. 3:**
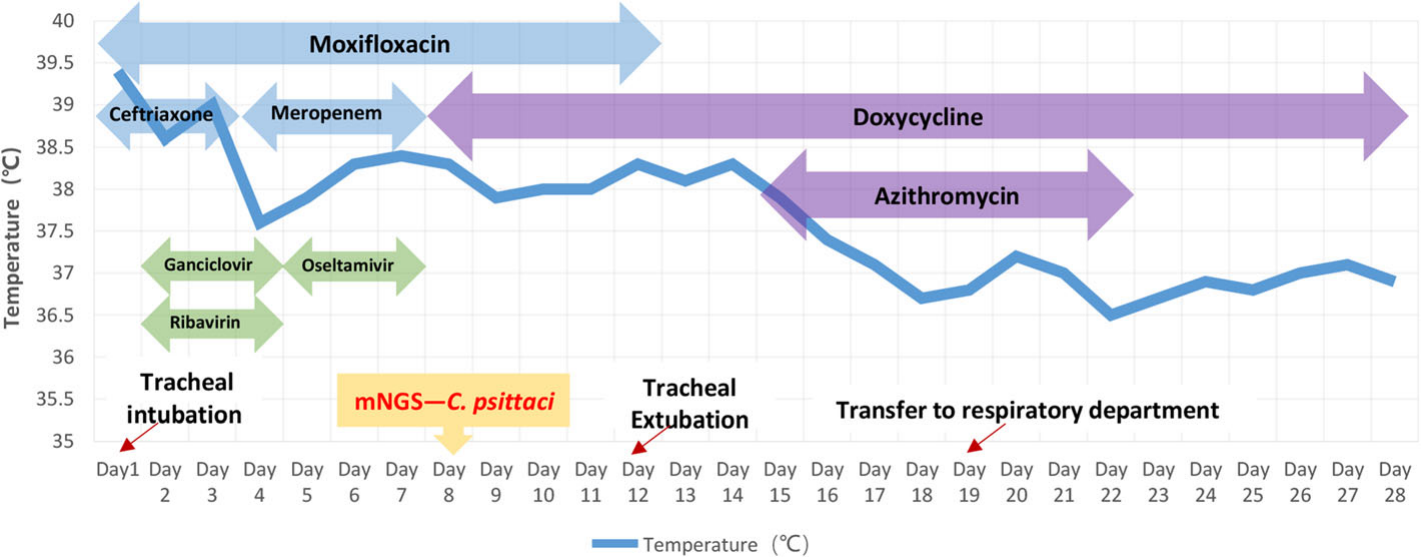
Body temperature and antimicrobial treatment during hospitalization Moxifloxacin 0.4 g qd (days 1–12), ceftriaxone 2.0 g qd (days 1–3), ganciclovir 0.25 g q12h (days 2–4), ribavirin 0.5 g q8h (days 2–4), meropenem 1.0 g q8h (days 4–7), oseltamivir 50 mg bid (days 5–7), doxycycline 0.2 g (day 8) and 0.1 g bid (days 8–28), azithromycin 0.5 g qd (days 15–21)

**Fig. 4:**
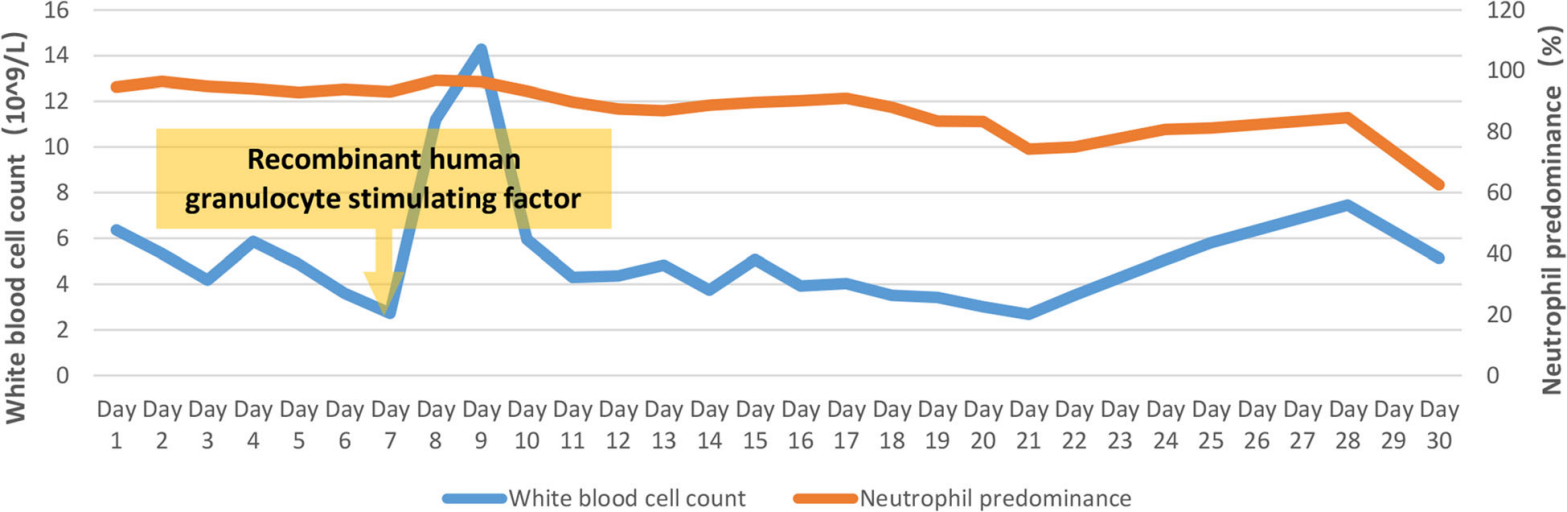
Change in white blood cell count and neutrophil predominance during hospitalization

**Fig. 5:**
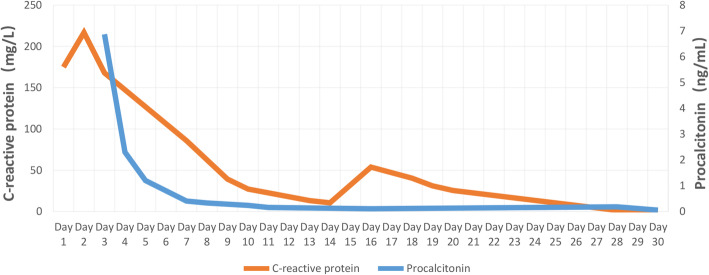
Change in C-reactive protein and procalcitonin during hospitalization

**Fig. 6:**
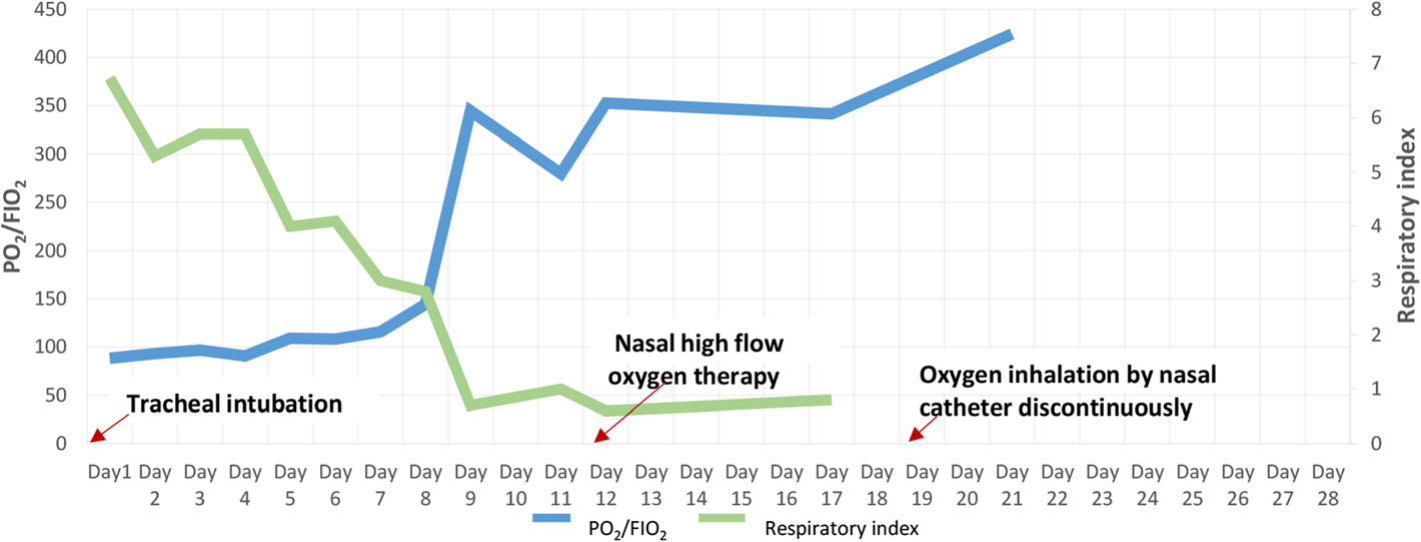
Change in oxygenation index and respiratory index during hospitalization

After admission, the patient was assessed with an acute physiology and chronic health evaluation (APACHE) II score of 14, sepsis related organ failure assessment (SOFA) score of 8 and mortality risk of 18.6%. She was given tracheal intubation, ventilator-assisted ventilation and anti-infective therapy, together with supportive treatment. Empirical antimicrobial and antiviral agents, including moxifloxacin, ceftriaxone, meropenem, ganciclovir, ribavirin and oseltamivir, were used initially. The peak temperature decreased, but the patient still had a fever. After mNGS of BALF and CSF suggested *C. psittaci* infection, antibacterial agents were switched to targeted doxycycline (0.2 g orally on day 8, then 0.1 g bid orally on days 8–28) combined with moxifloxacin (0.4 g qd intervenous drop infusion, days 8–12) and azithromycin (0.5 g qd intervenous drop infusion, days 15–21). Meropenem and empirical antiviral agents were ceased since there was no sequence read of bacteria or virus. Her fever was not completely relieved until the use of azithromycin, and finally returned to normal on day 16 after admission (Fig. 3). In accordance with temperature, the neutrophil predominance dropped continuously, along with CRP, PCT and interleukin (IL)-6 (Figs. 4 and 5). She was extubated successfully on day 12, and was given nasal high flow oxygen therapy after relief of ARDS (Fig. 6). Chest CT scan on day 14 showed multiple patchy shadows in both lungs, partial absorption in the right upper lobe, progressive exudation and consolidation in the remaining areas, and increased bilateral pleural effusions (Fig. 1B). The patient was transferred to the Respiratory Department (a general ward) on day 19. The patient’s chest CT re-examination on day 22 showed that the bilateral pneumonia was improved, the bilateral pleural effusions increased, and a small amount of pericardial effusion appeared (Fig. 1C). Because her condition improved daily, as well as the increase in oxygenation index (Fig. 6), she was discharged home on day 29.

The final diagnoses were made as psittacosis, severe community-acquired pneumonia, type I respiratory failure, acute respiratory distress syndrome (ARDS), meningitis, hypothyroidism, and chronic hepatitis B.

She returned on day 47 without complaint. No significant abnormalities were found in inflammatory indexes (PCT, CRP and IL-6) and liver function. Repeat chest CT showed that the exudation and consolidation of both lungs had almost disappeared, pleural effusion on the right side was reduced and the left pleural effusion had disappeared (Fig. 1D). The patient’s status returned close to premorbid condition on day 60 of follow-up.

## Discussion and conclusions

*C. psittaci*, is a Gram-negative, obligate intracellular bacterium that can cause serious psittacosis. Failure to diagnose the disease or identify the pathogen in time, with delayed use of effective antibiotics can cause high mortality in severe cases. Occasional outbreaks of psittacosis cause greater threat to society [2, 6]. Although the number of reported cases is increasing, and it has attracted more attention over the past decade, psittacosis is still regarded as an uncommon disease [7]. Due to low awareness and variable clinical presentation of the disease, psittacosis is often not recognized by clinicians, especially among general practitioners. So, it is necessary to discuss and review psittacosis to highlight clinical awareness and management of the disease.

Psittacosis mostly occurs after contact with contagious birds or feathers, excrement, or other substance of sick birds or poultry [4, 6, 8]. Besides, horses are demonstrated a novel source of infection [9, 10]. Human-to-human transmission has been reported but it is rare [6]. In this report, the patient denied direct or indirect contact with birds or poultry, nor there was a history of contact with horses. The probable source of the pathogen is unknown in this case.

Most infected people are adults aged 30–60 years, as was the present patient. There is an incubation period of 5–21 days before the symptoms appear. The clinical manifestations vary from asymptomatic infection to multiorgan disease, such as abortion in pregnant women, severe atypical pneumonia, or fatal meningitis in immunocompromised patients [3–5, 11, 12]. Our patient had most of the clinical manifestations outlined below. The onset symptoms usually appear abruptly with high fever, headache, chills, malaise, and myalgia. Mild dry cough can usually be found. Severe cases may involve breathing difficulty as with our patient. Chest pain and hemoptysis are uncommon. Severe headache is prominent in over a third of patients, which may be an indication of the occurrence and severity of meningitis. Fatal meningitis is a not uncommon and severe complication, which mainly manifests as severe headache and mental disorder [3, 4]. The majority of risk factors in meningitis are head trauma, upper respiratory infection and drug addiction [13]. Our patient had none of the risk factors. The typhoid-like illness with pulse temperature dissociation (fever without increased pulse rate), enlarged spleen, or nonspecific rash case can be sometimes present, although they were not seen in our case. A small number of patients also experience gastrointestinal symptoms such as vomiting, which was seen in our case. High APACHE II score, SOFA score and mortality risk indicated the severity of our patient when she suffered severe pneumonia and meningitis.

More than two thirds of patients’ leukocyte counts are within the normal range or at lower level during the acute phase. Inflammation indexes, including CRP, PCT and ESR etc. are elevated in initial blood tests, and are considered as objective markers for effectiveness of treatment, in addition to body temperature. Elevation of serum AST and ALT, which is related to liver injury, is present in some patients [4, 11]. These laboratory findings are not disease specific, as in the present case. As for the chronic hepatitis B in this case, because her liver function was normal during regular out-patient visit before the onset of psittacosis, which infers that elevation of serum AST and ALT was induced by infection of *C. psittaci,* it seems it’s not a predisposing factor. However, decreased T lymphocytes in this patient indicated that her immunity was suppressed at the onset of infection, which suggests *C. psittaci* may prone to cause infections in immunocompromised hosts. As we know, chronic liver disease could cause suppressed immunity. The possibility of chronic hepatitis B as a predisposing factor could not be eliminated entirely.

Although the radiographic features associated with psittacosis are not sufficiently characteristic to be distinguished from those of other types of CAP, they still show up specificity for atypical pneumonia. Abnormal chest X-rays are present in up to 90% of hospitalized cases. Chest radiography usually shows different degrees of exudation and consolidation, in which patchy shadows and reticular infiltrations are the most common manifestations. Large shadows on the lung lobes and extensive bilateral pneumonia may also appear in some severe cases, occasionally accompanied by pleural effusion. Chest lesions can be absorbed in 2–4 weeks after medical treatment [3, 4, 6, 7]. In this report, chest CT showed extensive bilateral pneumonia with pleural effusion, which was in accordance with severe cases.

The lack of a specific epidemiological history, clinical manifestation and radiographic features makes the diagnosis of psittacosis difficult, which may delay treatment, and sometimes even lead to serious outcome. Psittacosis pneumonia shares many features with CAP caused by viruses, fungi and some bacteria such as *Legionella*. They all can present with severe headache, high fever, rigors, dry cough and mental disorder, especially in severe cases [6, 14]. Therefore, the diagnosis of psittacosis needs to be determined by more specific detection [11, 15, 16]. Real-time polymerase chain reaction (PCR) has replaced culture and is regarded as the gold standard for detecting *C. psittaci* [7, 11, 15, 17]. However, PCR is more expensive and has not been developed for convenient use. mNGS can yield a high sensitivity for pathogen identification. mNGS for detection of *C. psittaci* by checking blood, BALF and CSF, etc. is applied clinically [18–21]. Under normal circumstance, there is no co-occurring or opportunistic pathogen in BALF and CSF because they both are aseptic. As we know, *C. psittaci* does not colonized in human body. Contamination of *C. psittaci* can be eliminated. As long as the process of sample taking, storage and transportation strictly follows aseptic procedures, contamination of other pathogens in BALF from higher airway or in CSF from skin can be eliminated too. mNGS of qualified BALF and CSF specimens help to make an etiologic diagnosis [19, 22]. In compendium of measure to control *C. psittaci* infection, additional diagnostic techniques such as genome sequencing are encouraged [6]. In our case, in spite of no definite history of contact with birds or poultry, *C. psittaci* in BALF and CSF was detected soon by mNGS following strict aseptic procedure and quality control, which supported the clinical diagnosis. There was no other evidence of fungus caused infection, one sequence read corresponding to *C. parapsilosis* in CSF was regarded as false positive. Accordingly, she received no antifungal agents. Besides, mNGS is less affected by prior antibiotic exposure, thereby emerging as a promising technique for detecting such complicated infectious disease [23, 24].

Tetracycline antibiotics are the first choice for treatment of human psittacosis. Mild to moderate psittacosis can be treated with doxycycline or minocycline orally, while severe disease needs to be treated with intravenous doxycycline. Generally, after treatment with tetracycline antibiotics, there is a response within 24–48 h, such as a decrease in body temperature. The course of medication should last at least 14 days, preferably up to 21 days, otherwise insufficient treatment will easily lead to relapse [4, 6, 11]. Macrolide antibiotics, such as azithromycin, are regarded as the best alternative for patients with contraindications to tetracyclines [12]. Fluoroquinolones, such as moxifloxacin and levofloxacin, have also been proven to be effective against *C. psittaci* [11, 25]. Severely infected patients in life-threatening condition may require combination treatment with tetracyclines, macrolides and quinolones. In present case, the patient achieved good results with targeted treatment of tetracycline orally combined with intravenous infusion of quinolones and macrolides. The corresponding effect and outcome after targeted treatment clinically supported the diagnosis of *C. psittaci* infection.

For this case, differential diagnosis of atypical pneumonia caused by other potential pathogens and autoimmune disease induced acute interstitial pneumonia was eliminated. There was no evidence in cytology and biochemistry of CSF supporting the existence of bacterial meningitis and tuberculosis meningitis. mNGS of BALF and CSF indicated *C. psittaci* infection. The favorable outcome responding to treatment supported the diagnosis of *C. psittaci* infection strongly. Based on the above clinical data, the etiologic diagnosis of psittacosis, *C. psittaci* caused severe pneumonia and meningitis was made.

In conclusion, when clinicians encounter patients with atypical pneumonia, accompanied by meningitis with symptom of headache or mental disorder, especially for cases with serious infections but lack response to conventional anti-infective therapy, psittacosis should be taken into consideration. mNGS is a promising detection method in such condition and is recommended.

At last, although it’s not sufficient enough for this case report to explore psittacosis and the role of mNGS in the diagnosis thoroughly, accumulation of such report is the need of the hour.

## Supplementary Information

**Additional file 1.**

## Data Availability

The datasets used and/or analysed during the current study are available from the corresponding author on reasonable request.

## Abbreviations

C. psittaci: Chlamydia psittaci
mNGS: Metagenome next-generation sequencing
BALF: Bronchoalveolar lavage fluid
CSF: Cerebrospinal fluid
CAP: Community-acquired pneumonia
MICU: Medical intensive care unit
FiO_2_: Fraction of inspired oxygen
WBC: White blood cell
CRP: C-reactive protein
PCT: Procalcitonin
ESR: Erythrocyte sedimentation rate
AST: Aspartate aminotransferase
ALT: Alanine aminotransferase
HBV: Hepatitis B virus
ADA: Adenosine deaminase
CT: Computed tomography
APACHE: Acute physiology and chronic health evaluation
SOFA: Sepsis related organ failure assessment
IL: Interleukin
PCR: Polymerase chain reaction
