# Supplementary material for: A case of chlamydia psittaci caused severe pneumonia and meningitis diagnosed by metagenome next-generation sequencing and clinical analysis: a case report and literature review
Source: BMC Infect Dis. 2021 Jun 30;21:621. doi: 10.1186/s12879-021-06205-5 (PMC8243071; doi:10.1186/s12879-021-06205-5)
Supplement: Supplementary file 1 — Additional file 1. [file 12879_2021_6205_MOESM1_ESM.docx]

**Additional file 1**

**Procedure and quality control of mNGS**

1. Procedure of mNGS

Bronchoalveolar lavage fluid (BALF) and cerebrospinal fluid (CSF) were collected by bronchoalveolar lavage and lumbar puncture, respectively. The samples were stored in a sterile container, then preserved and transported in dry ice. The whole process of sequencing and pathogen detection pipeline were carried out in the laboratory of Guangzhou Kingmed Medical Test Center Co.Ltd..

The DNA of samples was extracted by QIAsymphony Circulating NA Kit(Cus.48), together with a negative control (RNase-free water) and a positive control. Next, the DNA libraries were prepared using TruePrepTM DNA Library Prep Kit V2 for Illumina® (Vazyme Biotech Co., Ltd，TD503). The DNA libraries concentration was measured by Qubit. Then the sequencing was carried out by Illumina nextseq 500 system with 75 cycles Reagent Kit. High-quality sequencing data were obtained by filtering out reads of low-quality. Then, reads of human were removed by mapping reads to human reference genome. The remaining data were aligned to the microbial genome database.

The procuture of filtering, mapping and alignment are carriyed out by the widely used software Sequence-Based Ultra-Rapid Pathogen Identification (SURPI), which is generally considered to be highly accurate. The microbial genome database is MetagenomicX for clinical application. The whole database contains 22934 microorganisms, which covers most of the microbial genomes which have been sequenced. 8704 of the total microorganisms in the first-grade database have integral sequence of whole genome and detailed clinical analysis, which covers most of the known pathogenic bacteria, viruses, fungi and parasites. This first-grade database with high-quality genomes is used as first choice.

# 2. Quality control of procedure

During the process of sampling, storage, transportation and sequencing, aseptic procedures are strictly followed to ensure that qualified BALF and CSF specimens are collected. We collected the middle part of the BALF and CSF. In the lab, the specimens were kept in the -80 degree refrigerator to prevent nucleic acid degradation. All detection procedures are finished within 48 hours after receiving specimen ensuring timely guidance for clinical treatment.

Both Nucleic acid extraction and library preparation were conducted in parallel with quality control samples. And we compared the results of this whole process with the results analysed by software of Burrows Wheeler Alignment (BWA). The results of the two pipelines are highly consistent. To eliminate background interference, a minimum threshold of 10 RPM-r (RPM defined as Reads per million, RPM-r defined as RPMsample/RPMno-template-control) is designated for reporting the detection of a microorganism as “detected”.

For these two mNGS of BALF and CSF, 99.24% and 99.52% reads were filtered for human genome and respectively, and 79722 reads and 93308 reads were mapped to the microbial genomes database respectively. The Q30 were 92.13% and 94.26% respectively. The quality scores across all bases in the mNGS of BALF and CSF were showed as the figures below.


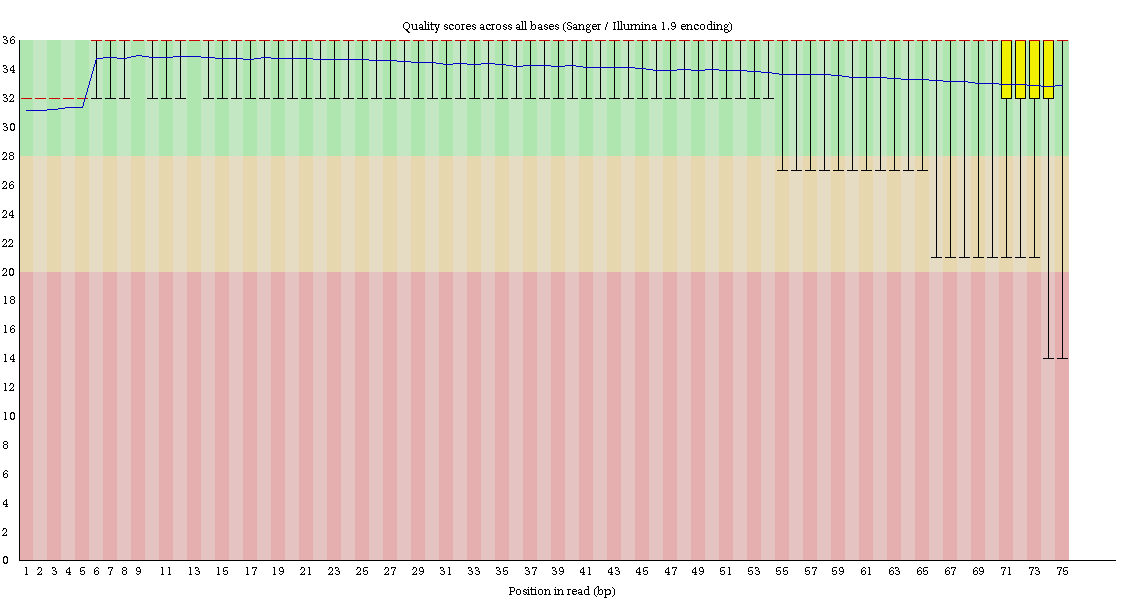


Fig.1 The quality scores across all bases in the mNGS of BALF


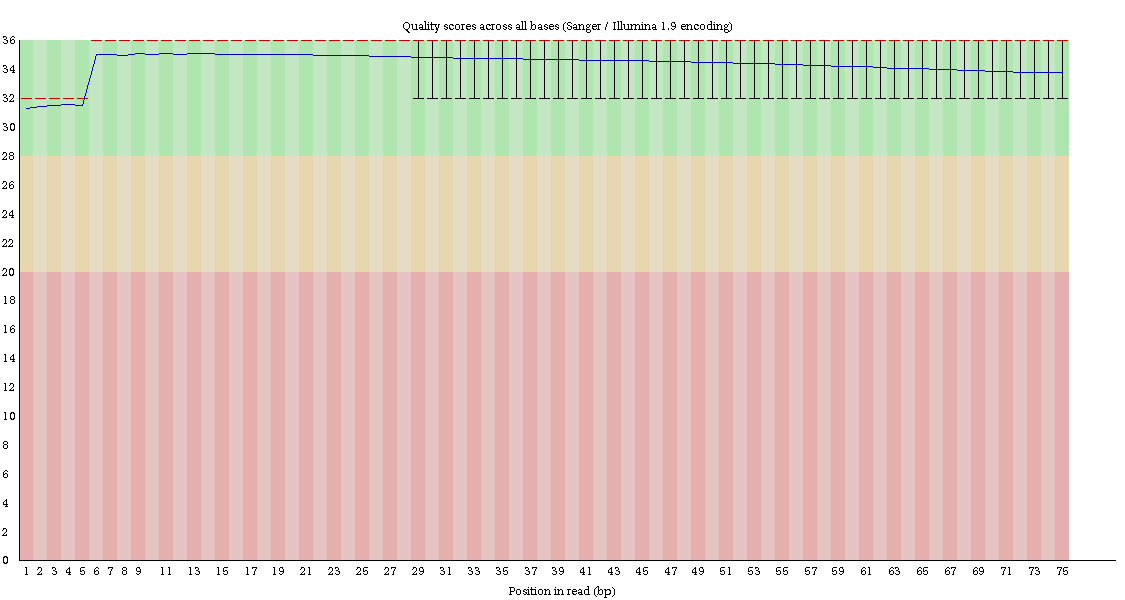


Fig. 2 The quality scores across all bases in the mNGS of CSF
